# The effect of school size and class size on school preparedness

**DOI:** 10.3389/fpsyg.2024.1354072

**Published:** 2024-02-26

**Authors:** Faye Antoniou, Mohammed H. Alghamdi, Kosuke Kawai

**Affiliations:** ^1^Department of Educational Studies, National and Kapodistrian University of Athens, Athens, Greece; ^2^Department of Self-Development Skills, King Saud University, Riyadh, Saudi Arabia; ^3^David Geffen School of Medicine, University of California Los Angeles, Los Angeles, CA, United States

**Keywords:** cusp catastrophe model, NLDPS, school readiness, PISA 2018, school size, class size

## Abstract

The purpose of the present study was to understand students’ school readiness as a function of student and teacher behaviors but also school size and class size using both linear and non-linear analytical approaches. Data came from 21,903 schools distributed across 80 countries as per the 2018 cohort of the PISA database. Results pointed to a preference for the Cusp model in that the relationship between school and class sizes with achievement proved to be best described by the non-linearity of the Cusp catastrophe model. The critical benchmarks were a school size of 801 students and a class size of 27 students for which increases beyond those thresholds were linked to nonlinearity and unpredictability in school readiness. For this reason, we suggest using the cusp catastrophe model from Nonlinear Dynamical Systems Theory (NDST) to understand more fully such complex phenomena.

## 1 The effect of school size and class size on school preparedness

It’s important to note that a school’s size and course offerings greatly affect academic performance. A smaller class size allows the instructor to deliver a more tailored education, which increases the likelihood of meeting each student’s requirements and concerns. This may create a learning environment where students feel recognized and understood, which will boost their engagement and drive (Ajami and Akinyele, 2014). Teachers in smaller courses are also better at using a variety of instructional strategies, accommodating different learning modalities, and creating dynamic and interesting learning environments (Bradley and Taylor, 1998). Since there are fewer students to watch in a classroom, keeping discipline is easier, which may reduce disturbances and improve learning. This is shown by greater results on standardized examinations as well as more positive long-term educational outcomes (Mosteller, 1995; Tseng, 2010; Krassel and Heinesen, 2014; Gereshenson and Langbein, 2015; Lowenthal et al., 2019). According to Blatchford et al. (2011), Nandrup (2016), and Yamamori et al. (2021), the number of pupils in a class has a considerable impact on both the educational experience students have and the academic results they attain.

Class size and the ratio of staff to students are often used as measures to evaluate the quality of higher education (Thom, 1975; Molenaar and Oppenheimer, 1985; Brown, 1995; Bandiera et al., 2010; Martin 2015; Konstantopoulos and Shen, 2023). These studies were conducted by Stewart and Peregoy, 1983; Guastello, 1984, 1987,1992; Bandiera et al. (2010); Konstantopoulos and Shen (2023), and Martin (2015). Some studies suggest that larger class sizes hurt student learning; however, a significant number of studies present findings that are inconclusive or demonstrate a combination of positive and negative effects (Bellante, 1972; Edgell, 1981; Hancock, 1996; Kennedy and Siegfried, 1997; Hill, 1998; Jarvis, 2007; Gleason, 2012; De Paola et al., 2013; Matta et al., 2015; Olson et al., 2011). However, previous research has shown that students tend to have a more positive perception of their learning experience when the number of classes they are required to attend is decreased (Kwan, 1999; Van der Maas and Molennar, 1992; Bedard and Kuhn, 2008; Westerlund, 2008; Mandel and Sussmuth, 2011; Monks and Schmidt, 2011; Benton and Cashin, 2012; Sapelli and Illanes, 2016; Hufford et al., 2003; Stamovlasis, 2006). The use of active learning strategies by teachers in smaller class sizes, in addition to the provision of more individualized attention to students (Lammers and Murphy, 2002; Arias and Walker, 2004; Kokkelenberg et al., 2006; Monks and Schmidt, 2011; Van der Maas et al., 2003), could be the reason for this phenomenon (Lammers and Murphy, 2002; Arias and Walker, 2004; Kokkelenberg et al., 2006; Monks and Schmidt, 2011). However, the amount of material that is currently available about the challenges that are related to the application of active learning approaches in smaller class sizes is quite limited (Wright et al., 2017).

A large, influential study namely, the Student Teacher Achievement Ratio (STAR) study reported significant benefits from class size reductions on students’ achievement, if these reductions take place early with the effects being more pronounced for students from disadvantaged family backgrounds (Word et al., 1990; Mosteller, 1995; Finn and Achilles, 1999; Krueger, 1999; Nye et al., 2000). These findings are backed up by several academic publications, such as those written by Guastello (2001), Word et al. (1990), Mosteller (1995), Finn and Achilles (1999), Krueger (1999), and, Nye et al. (2000), amongst others. Throughout the early years of their schooling, policymakers need to reflect on the most effective way to direct Corporate Social Responsibility (CSR) programs toward children who are starting in life with socioeconomic disadvantages. Policymakers also have the option of choosing to offer funding for CSR programs while at the same time providing local school leaders the liberty to decide how such programs will be implemented. It is essential to take into consideration a cost–benefit analysis of educational policy whenever one is charged with making judgments about the maximum number of students allowed in a given classroom.

### 1.1 Evaluating the type of relationship between school size, class size, and school outcomes

Past studies have primarily engaged linear modeling to evaluate the role of class size on school outcomes. The idea that the relationship between class size and student, teacher, and school outcomes is linear falls short for the following reasons. Linear models operate under the assumption that the relationship between variables is best depicted by a mathematical straight line. However, human behavior and outcomes, such as learning and teaching, are inherently complex and multi-faceted. A simple linear relationship might not capture all the nuances and intricacies involved.

Empirical evidence has reported both linear and non-linear effects using, for example, quadratic models. The problem with those findings is that they reported both positive and negative nonlinear trajectories that contradict each other (e.g., Foreman-Peck and Foreman-Peck, 2006; Crispin, 2016). It is possible, however, that the impact of class size on outcomes changes when a certain threshold is crossed. For example, reducing a class from 40 to 30 students might have a more significant impact than reducing it from 30 to 20. Such an effect was reported in the Lee and Smith (1997) study as they reported that school sizes ranging in number of students from 600 and 900 were optimal in facilitating reading and math outcomes. Linear models would most likely be ineffective in capturing such non-linear effects. For the above, more elaborate, and complex analytical models that take into account non-linear changes in behavior and operate using multiple predictors who may exert linear and nonlinear effects are needed. Empirical evidence of this effect has been provided by Cobb et al., 1983; Cobb and Zacks, 1985; Bowne et al. (2017) who reported that “both class size and child-teacher ratio showed nonlinear relationships with cognitive and achievement effect sizes” (p. 407) with implications that this relationship may be further complicated by SES associations. In a large-scale study by Cobb, 1981; Lee and Loeb (2000), concerns were raised about the hypothesized relationship between school size and student learning. In the present study, we propose that the relationship between class size and school outcomes becomes non-linear following a crucial threshold in class size, beyond which, student and school outcomes become unpredictable and chaotic (Oliva et al., 1987). This is why the cusp model may provide the most appropriate means to evaluate the proposed relationships.

### 1.2 Nonlinear dynamical systems theory and the cusp catastrophe

The cusp catastrophe model is essential in nonlinear dynamic systems theory for explaining sudden and discontinuous system state changes generated by continuous variables. The model works across fields with more recent applications in psychology, education, medicine, and public health. It efficiently handles complex linear and nonlinear interactions between independent variables (Chen and Chen, 2017) toward the understanding of behavioral phenomena. As control variables change, the cusp model can decipher abrupt and sudden behavioral changes offering insights into the stability and transitioning of human behavior (Chen et al., 2014). Applications in the field of education include the investigation of cognitive functioning (Stamovlasis, 2011; Tsitsipis et al., 2012), the teaching of physics (Papageorgiou et al., 2010), and chemistry (Stamovlasis et al., 2005). In the examination of stress and trauma the cusp model has contributed significantly to our understanding of changes in human emotions as individuals transition from one psychological state to another (Kira et al., 2019, 2020). Thus, the cusp catastrophe model has recently been popularized in the social sciences to examine scenarios where shuttle changes in a parameter are associated with drastic and dramatic changes in outcome variables The model employs a potential function, *f* (y; a, b) for a single dependent variable y given linear and nonlinear parameters a and b:

*f* (y; a, b) = ay + 1/2by^2^-1/4y^4^ (Eq. 1).

**Figure 1:**
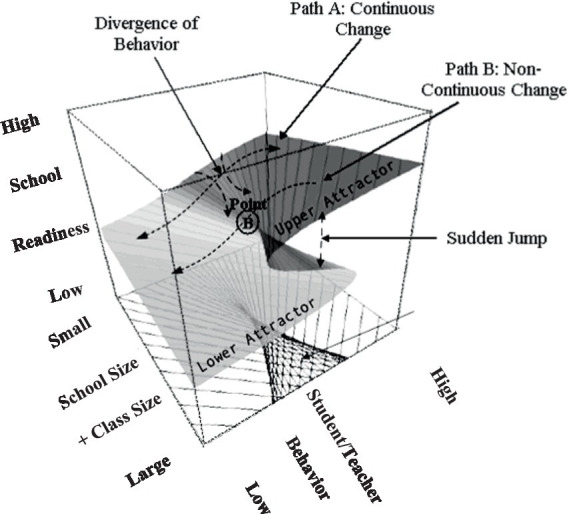
Description of the cusp model with the outcome variable school readiness; it was predicted linearly using the asymmetry terms of student and teacher behaviors and non-linearly using the bifurcation terms of school size and class size (Tasker, 2003).

The phenomenon under study is termed the “catastrophe set” which evaluates outcomes inside the parameter space of coordinates (a, b). As shown in Figure 1, when the “b” terms (bifurcation variables), in our instance school size and class size are at low levels changes in school readiness are expected to be linear and smooth, likely fitting the premises of the linear model (see expected Pattern A to linearity). When, however, increases in school size and/or class size exceed a worrisome, critical level, school readiness oscillates between two behavioral modes, low and high readiness, reaching a state of unpredictability (see Pattern B to non-linearity). Point B in the figure is termed “the cusp point” and reflects unpredictable changes in the outcome variable following midpoint levels in the bifurcation variable. In other words when schools and class sizes are small and move toward medium levels, school readiness is expected to covary in a linear manner; this linear prediction is disturbed following some critical levels of both school size and class size suggesting that increases in these variables are no longer adaptive.

The purpose of the present study was to understand students’ school readiness as a function of student and teacher behaviors but also school size and class size using both linear and non-linear data analysis procedures to understand more fully such complex relations.

## 2 Method

### 2.1 Participants and procedures

Data came from 21,903 schools distributed across 80 countries as per the 2018 cohort of the PISA database (OECD, 2019). The unit of analysis was the school. Thus, student and teacher estimates were aggregated per school. Student participants in PISA 2018 had to be in the range of 15 years 3 months and 16 years and 2 months and had to be in grade 7 or above. Procedures regarding ethics and sample selection are described here (https://www.oecd.org/pisa/publications/pisa-2018-results.htm). Data may be accessed at https://www.oecd.org/pisa/data/.

### 2.2 Measures

All measures were derived from the PISA 2018 most recent cohort.

#### 2.2.1 School readiness

This scale is comprised of 8 items completed by school principals on factors that hinder a school’s capacity to provide instruction. The items use the stem: “Is your school’s capacity to provide instruction hindered by any of the following issues” with the content relating to the shortage of teaching staff, insufficient instructional materials, inadequate physical infrastructure, poor lab equipment, and shortage of ICT resources (Werblow and Duesbery, 2009; Wobmann and West, 2006). Scaling ranged between “not at all” and “a lot” (see Supplementary Appendix A1). Given the high internal consistency reliability of the items with omega being at 0.839, factor scores were estimated using maximum likelihood.

#### 2.2.2 Student behavior hindering learning

It was assessed using 3 student-reported items that evaluate how often students disrupt lessons (a) with noise and talking, (b) with misbehavior, and (c) by being late or absent (Alexander et al., 1992). The items are scored using a 4-point scaling system anchored between “never” and “almost every day.” Scores were estimated using Weighted Likelihood Estimation (WLE).

#### 2.2.3 Teacher behavior hindering learning

A 4-item scale was created using student responses to assess how often the teacher (a) explains things in a way that is difficult to understand, (b) does not give enough help when you need it, (c) does not seem to care about whether or not you learn, and (d) does not keep order in the classroom. Items engage a 4-point scaling system from “never” to “almost every day.” Estimated factor scores utilized the WLE estimator.

#### 2.2.4 Class size and school size

Class size reflected the number of students in the class using a categorically ordered variable with intervals of 5 students. The categories ranged between “fewer than 15 students” to “more than 50 students.” School size was measured as a summative variable expressing the number of students in the school. It is a measure of total enrollment rather than expressing estimates for particular grades, cohorts, genders, or else.

### 2.3 Statistical data analyses

#### 2.3.1 Cusp catastrophe

At present, there exist multiple analytical models that can be utilized for the detection of a cusp catastrophe. Among these models, Cobb’s (1998) methodology and its implementation in R using Grasman’s cusp package (Grasman et al., 2009) are widely recognized and accepted. Additionally, the polynomial regression model proposed by Guastello (2002) and the modifications made by Chen et al. (2020) to Cobb’s method are also noteworthy alternatives although limited by the unavailability of routines in statistical packages. While both Cobb’s and Guastello’s models have been widely used, we consider Cobb’s (1981) method as more closely associated with catastrophe theory whereas Guastello’s methodology is more general and includes various forms of non-linear regression models, such as the quadratic. Thus, we choose the methodology proposed by Cobb (1998), which gained popularity through its implementation in R. To achieve optimality, several conditions must be satisfied by the model. Firstly, the asymmetry and bifurcation parameters should exhibit a significant effect. Secondly, the cusp model should demonstrate superiority over linear and non-linear competing models such as the logistic, the quadratic, and the cusp. Third, a relatively small proportion of observations, approximately 10%, should fall within the bifurcation area. Lastly, there should be evidence indicating the presence of bimodality within the bifurcation area and multimodality elsewhere for the outcome variable. We deferred using the pseudo-R-squared statistic provided by the package given that it can take negative values. Of note here is the sign of the bifurcation term(s) which requires additional elaboration. Assuming that the bifurcation term is scaled so that higher scores are indicative of unpredictability, then a positive coefficient should be observed. A positive coefficient indicates that the relationship between bifurcation and the outcome variable is linear at low levels of the bifurcation term and becomes non-linear later on. Based on that, in the present study, a positive slope in the bifurcation term was desirable. On the contrary, a negative bifurcation term suggests that at low levels of the splitting factor/bifurcation term, the system is chaotic, and as the scores on the splitting factor increase, linearity, and equilibrium are gradually present. This is not the case in the present study for which unpredictability is expected when increases in school and class size move beyond some adaptive level.

## 3 Results

### 3.1 Prerequisite assumptions of cusp catastrophe

**Figure 2:**
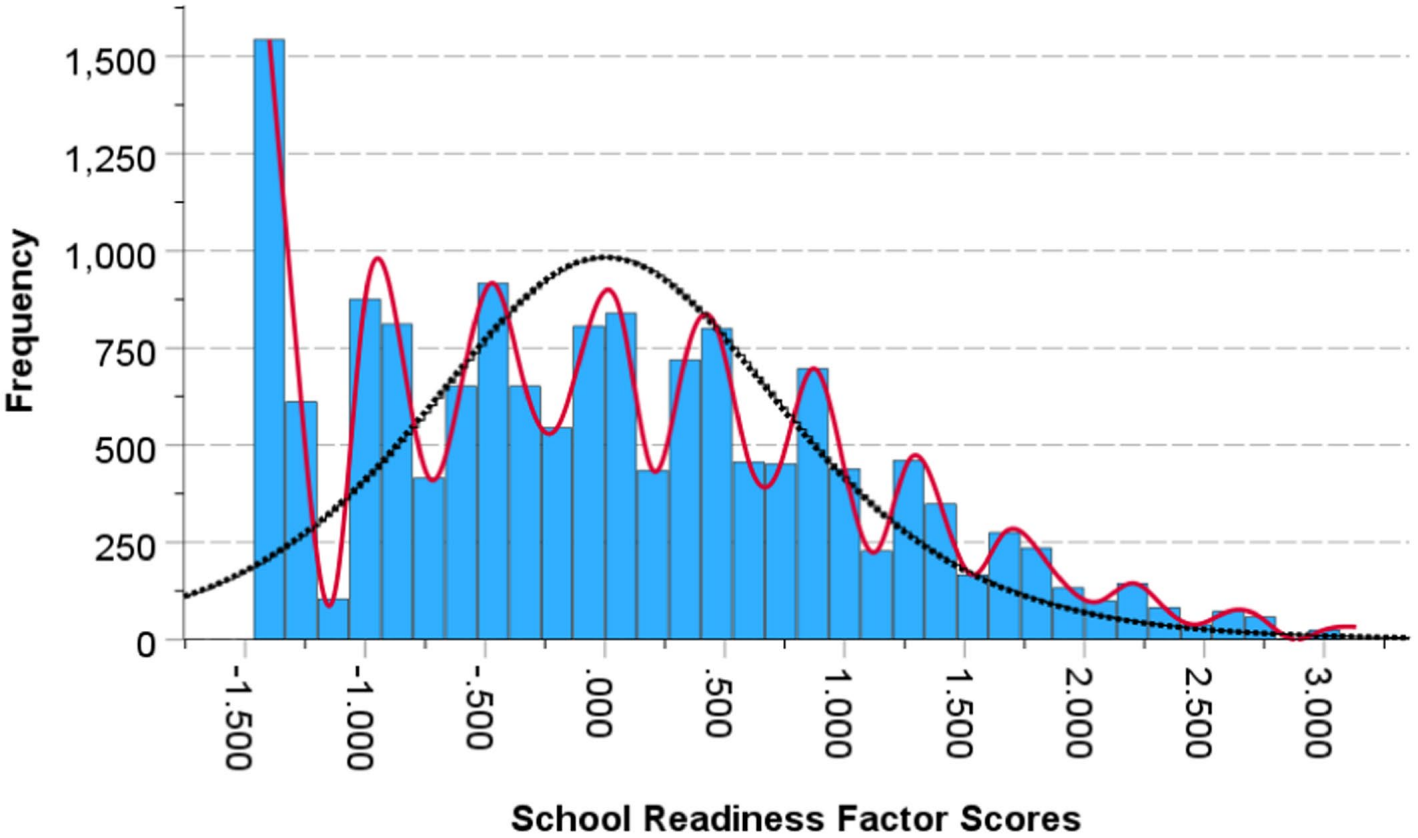
Plotting the multimodal distribution of the dependent variable school readiness.

**Figure 3:**
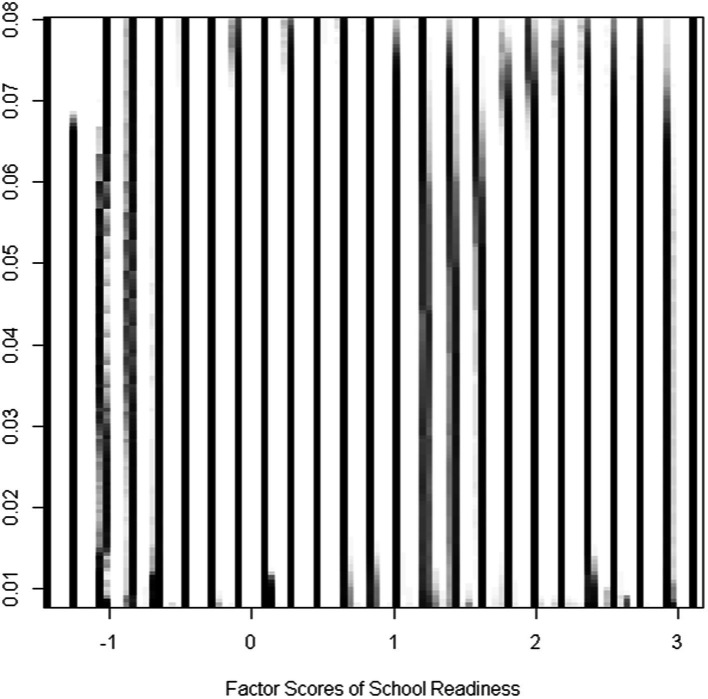
Mode locations for each bandwidth of the school readiness factor scores construct. The number of modes reflects those shown in Figure 2.

One of the important assumptions of the cusp catastrophe is that the dependent variable must have more than one mode. In the present study, the latent school readiness variable presented itself with multimodality as shown in Figure 2 satisfying the prerequisites of the cusp model. Further evidence of the multimodality of school readiness is shown in Figure 3 using the mode tree (Minnotte and Scott, 1993). The figure displays the mode locations of the readiness variable for each bandwidth. The figure displays 25 modes suggesting multimodality as does Figure 2.

### 3.2 Predicting school readiness from student and teacher behaviors linearly and from school and class size nonlinearly

**Figure 4:**
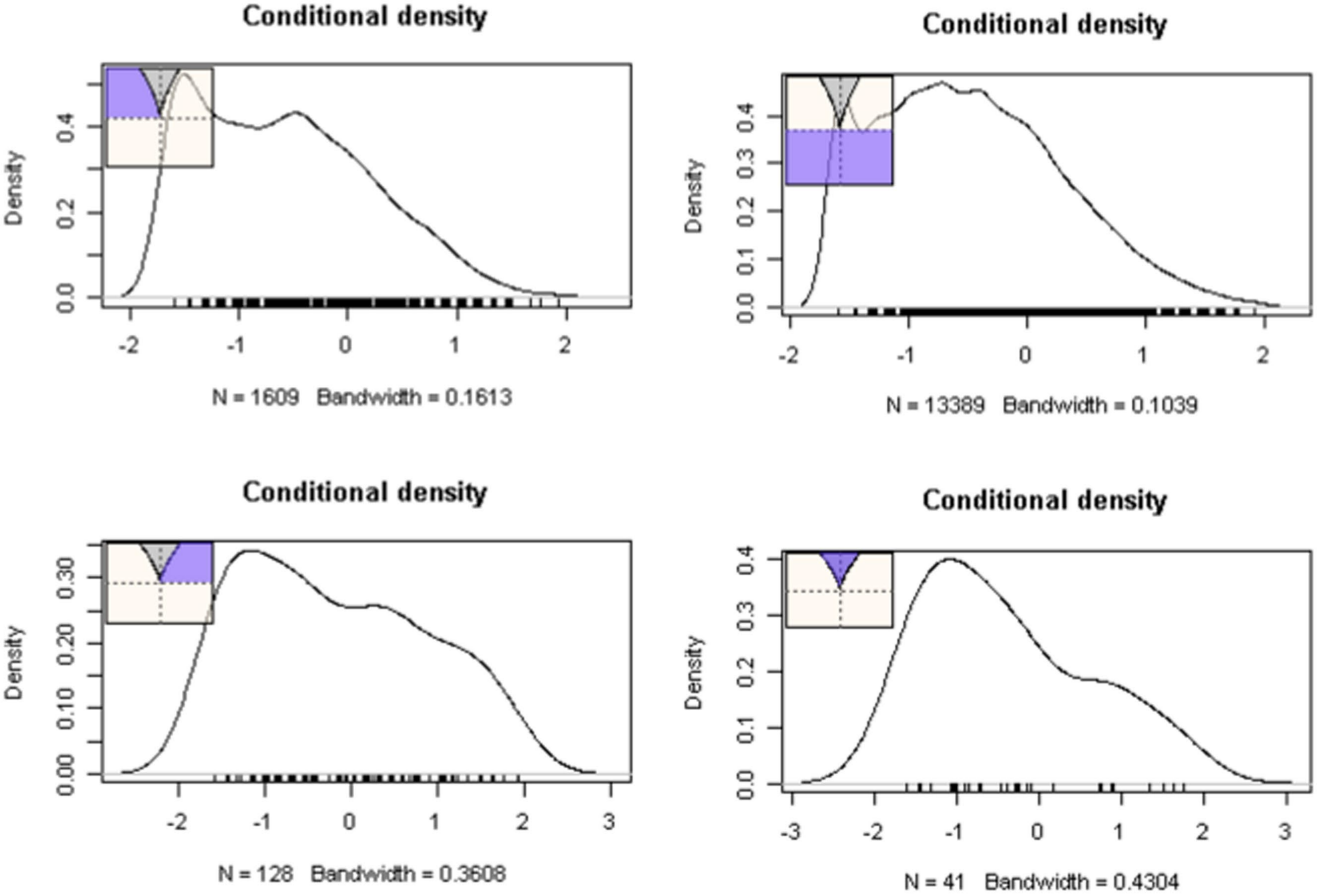
Frequency of observations at various places on the response surface as indicated by the shaded areas. The presence of skew and multimodality are apparent in several areas of the lower response surface.

**Figure 5:**
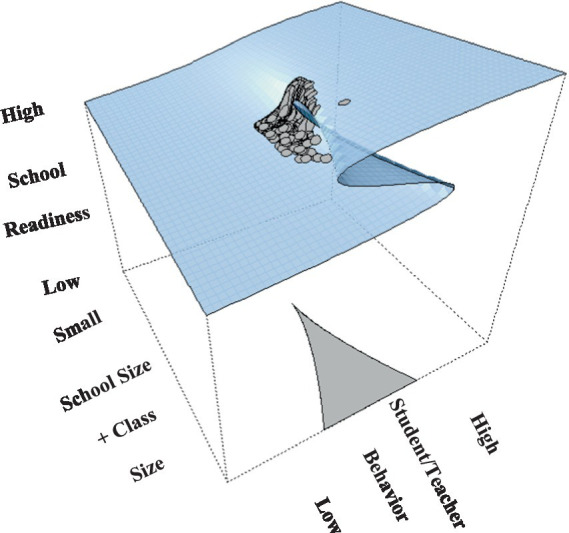
Cusp catastrophe upper and lower surfaces with observations transitioning between the two and within the bifurcation area.

**Figure 6:**
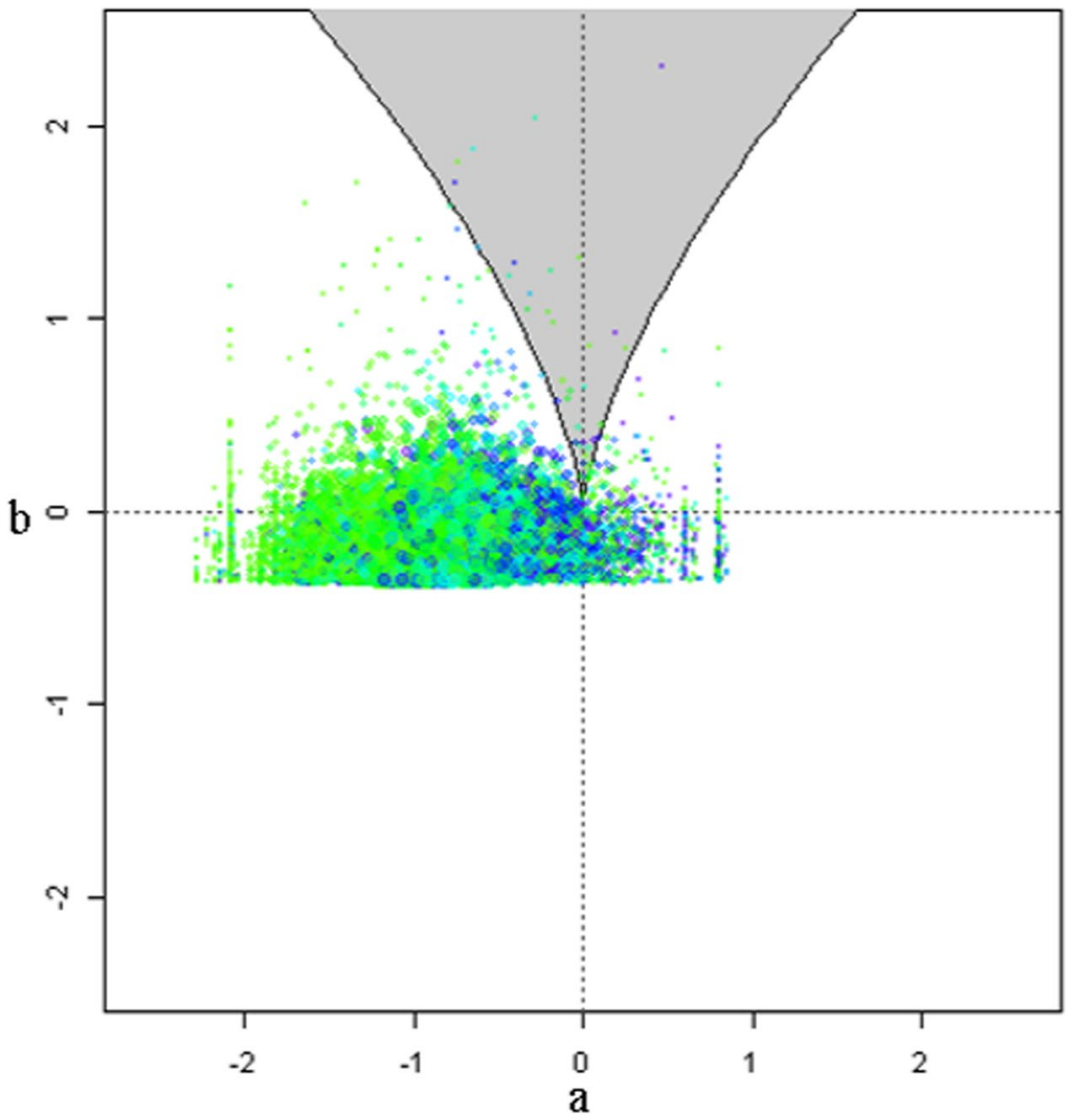
Control plane scatterplot with darker dots showing placement of observations close to the upper surface and light color dots towards the lower surface.

Intercept and slope terms of the cusp model are shown in Table 1 with all terms being significant. School readiness was positively predicted by the linear contribution of student and teacher behaviors (*b*_Student_ = 0.196, *p* < 0.001; *b*_Teacher_ = 0.264, *p* < 0.001). Interestingly, both bifurcation terms were also significant signaling non-linearity. Specifically, as school size and class size increase beyond a specific critical threshold, their relationship to school readiness becomes chaotic and unpredictable (*b*_School size_ = 0.001, *p* < 0.001; *b*_Class size_ = 0.004, *p* = 0.027). The critical benchmarks were a school size of 801 students and a class size of 27 students for which increases beyond those thresholds were linked to non-linearity and unpredictability in school readiness. When testing optimal model fit between linear and non-linear models (see Table 2) results pointed to the superiority of the cusp model using the information criteria values of AIC, AICc, and BIC over the competing models (a) linear, (b) quadratic, and (c) logistic, which were consistently lower in the cusp model compared to all other models. Furthermore, a chi-square test contrasted linear and cusp models pointed to a significant misfit of the linear model [*χ*^2^(2) = 1732, *p* < 0.001]. Further evidence for the cusp model’s preference is shown in Figure 4, with multimodal distributions at various areas across the response surface and bimodality within the bifurcation area (bottom right figure). Visually speaking, Figure 5 displays the observations as they oscillate between upper and lower surfaces and within the bifurcation area again fitting the expectations of the cusp catastrophe model. An ancillary to Figure 5, is Figure 6, which displays the relative position of the observations to the upper and lower surfaces using a control plane scatterplot. Observations with darker colors (e.g., purple) are positioned closer to the upper surface and those with lighter colors (e.g., light green) are closer to the lower surface (see Grasman et al., 2009).

**Table 1:** Parameter estimates of the cusp model for the prediction of school readiness using a combination of asymmetry (student and teacher behaviors) and bifurcation (school and class size) predictors.

Cusp model intercept and slope terms	Unstandardized B	S.E.	*Z*-test	Value of *p*
a (Intercept)	−0.870	0.027	−31.986	<0.001***
a_1_ (Student behavior)	0.196	0.012	15.911	<0.001***
a_2_ (Teacher behavior)	0.264	0.019	13.803	<0.001***
b (Intercept)	−0.421	0.077	−5.468	<0.001***
b_1_ (School size)	0.001	0.001	18.933	<0.001***
b_2_ (Class size)	0.004	0.001	2.212	0.027*
w (Intercept)	−0.496	0.009	−51.496	<0.001***
w (School readiness)	0.770	0.004	173.993	<0.001***

Note: The terms a, b, and w refer to asymmetry, bifurcation, and outcome variables’ intercept terms with those including a subscript the specific slopes for the asymmetry and bifurcation variables. ***p <. 001; **p < 0.01; *p < 0.05.

**Table 2:** Comparing linear and cusp models using information criteria.

Models tested	Loglikelihood	Par	AIC	AICc	BIC
1. Linear	−20651.560	6	41315.11	41315.12	41360.87
2. Logistic	−20579.590	7	41173.18	41173.18	41226.56
3. Quadratic	−20629.389	8	41274.78	41310.37	41335.79
4. Cusp	−19784.772	9	39587.55	39587.56	39656.19

Par, number of freely estimated parameters; AIC, akaike criterion; AICc, corrected AIC; BIC, Bayesian information criterion. Preference is given to the BIC. Its sample-adjusted variant is not included because of the large sample size of the PISA 2018 cohort.

## 4 Discussion

The purpose of the present study was to understand students’ school readiness as a function of student and teacher behaviors but also school size and class size using both linear and non-linear analytical approaches. Results pointed to a preference for the cusp catastrophe model in that the relationship between school and class size with achievement is determined by specific thresholds of these variables.

Past research indicates that the size of a class has a significant influence on the academic achievement of students. The work by Kenayathulla et al. (2019) favored the role and functioning of smaller classrooms and their positive impact on academic achievement. Studies on the Portugal Programme Mais Sucesso Escolar (PMSE) indicate that factors such as class size, composition, and tailored instruction might lead to a decrease in grade repetition and an enhancement in academic achievement (Barata et al., 2015). Nevertheless, these findings have contentious ramifications for educational policy. Other studies on the other hand (e.g., Filges et al., 2018), pointed out that decreasing class sizes has minimal impact and that there are more cost-effective methods for enhancing student achievement. These unequivocal findings demonstrate that class size and outcomes vary based on the circumstances and composition of the student population (Milesi and Gamoran, 2006).

The most important finding of the present study was that preference for the cusp model allowed us to identify important thresholds for which student readiness is no longer predictable. These thresholds were 801 students for school size and 27 students for class size. Interestingly, the estimates for school size agree with earlier suggestions using quadratic models suggesting that between 600 and 900 students is the optimal school size (Lee and Smith, 1997) and also the work of Andrews et al. (2002) who reported dysfunctional schools when exceeding 1,000 students. For class size, earlier work suggested diminishing returns in that reducing class size from 30 to 25 students is more beneficial compared to reducing it from 20 to 15 (Mosteller, 1995; Krueger, 1999). Thus, the currently identified threshold falls within earlier predictions (Word et al., 1990; Finn and Achilles, 1999; Nye et al., 2000).

### 4.1 Study implications for educational policy

The growing body of research suggesting that larger school and class sizes harm student achievement requires a thorough reassessment of educational systems. When class and school sizes become too large, the amount of attention given to each student decreases, which can hinder customized instruction and result in a decrease in academic performance (Blatchford, 2003; Hattie, 2006). This issue highlights the necessity for policymakers to adopt initiatives focused on maintaining or decreasing class and school sizes to cultivate more efficient learning environments. Possible approaches could involve implementing strict class size restrictions, especially in early schooling where personalized attention is vital, and reorganizing bigger educational institutions into smaller learning communities to improve individualization and assistance (Lee and Smith, 1997). Furthermore, it is important to implement laws that provide fair and equal access to small-sized classrooms and schools among all socioeconomic and demographic groups. This will help to resolve any potential inequalities in educational achievements. Furthermore, a transition to smaller educational environments requires corresponding improvements in teacher recruiting, training, and retention methods, guaranteeing that the standard of education remains uncompromised. In conclusion, although there are difficulties in managing and funding efforts to optimize class and school sizes, the possibility of achieving substantial enhancements in student performance makes it an essential area of concentration for educational reform and policy formulation.

### 4.2 Study limitations and future directions

The variables “school size,” and “classroom size” have long been recognized as significant in research although past studies have presented several methodological and design deficiencies. The existing research on the functioning of school and classroom size has several limitations. Firstly, it lacks generalizability due to the impracticality of randomly assigning students to schools and classes advising caution before generalizing the present findings beyond the specific sample. Secondly, there is a lack of consistent measures, particularly in quantifying the distinction between “large” and “small” schools with proxy measurements including the number of teachers, the number of students, or other ratio variables, which are also challenged by the violation of distributional assumptions. There is also a need to engage multivariate analyses that can consider the variations in sampling across students and schools using both linear and non-linear means. In the present study, the use of international population data using rigorous procedures for sampling and representativeness in each country overcomes one of the major limitations of past studies. Furthermore, the analytical framework utilized here is not without limitations. The cusp catastrophe model has been criticized as overfitting the data, thus, limiting model generality (Poston and Stewart, 1978). Concerns about the reliability of the findings from small samples have also been raised (Zhang, 2016) as well as the model’s generality to real-world phenomena (Schelling, 1973). Others were concerned that the complexity and uncertainty of real-world phenomena cannot be captured by a set of mathematical equations (Borsboom and Cramer, 2013) and specifically one type of asymmetry measured within the cusp catastrophe model (Cramer, 2008).

In the future, we advise the use of the present analytical framework using a per-country analysis as well as the invariance of the findings across important moderating variables such as gender, SES, urbanicity, private or public schooling, and other variables which were found to be important predictors of a school’s climate.

## 5 Conclusion

In this comprehensive examination of the factors influencing students’ school readiness, a significant discovery emerged: the relationship between school and class sizes with achievement is best described by the non-linear complexities of the Cusp catastrophe model. This study, utilizing data from over 21,000 schools across 80 countries, revealed critical thresholds at a school size of 801 students and a class size of 27 students. Beyond these points, size increases are associated with unpredictability and decreased school readiness. This suggests a pronounced shift in the traditional understanding of educational environments, emphasizing the importance of maintaining optimal class and school sizes to ensure effective learning and teaching. The findings underscore the need for policymakers to reconsider current educational structures, advocating for more personalized and manageable learning environments to enhance student achievement. While this study provides a groundbreaking insight into the dynamics of educational settings, its reliance on the cusp catastrophe model and the specific thresholds identified necessitates further investigation and validation to ensure widespread applicability and understanding of its implications in the complex landscape of educational policy and practice.

## Data availability statement

Publicly available datasets were analyzed in this study. This data can be found at: https://www.oecd.org/pisa/data/2018database/.

## Ethics statement

Ethical review and approval was not required for the study on human participants in accordance with the local legislation and institutional requirements. Written informed consent from the patients/ participants or patients/participants legal guardian/next of kin was not required to participate in this study in accordance with the national legislation and the institutional requirements.

## Author contributions

FA: Conceptualization, Investigation, Methodology, Writing – original draft, Writing – review & editing. MA: Data curation, Funding acquisition, Investigation, Writing – review & editing. KK: Formal analysis, Methodology, Software, Writing – review & editing.
