# Supplementary material for: The effect of school size and class size on school preparedness
Source: Front Psychol. 2024 Feb 26;15:1354072. doi: 10.3389/fpsyg.2024.1354072 (PMC11002959; doi:10.3389/fpsyg.2024.1354072)
Supplement: Supplementary file 1 [file Table_1.docx]

**Appendices**

**Appendix A**

Outcome Variable “School Readiness” Implemented in the PISA 2018 Cohort.


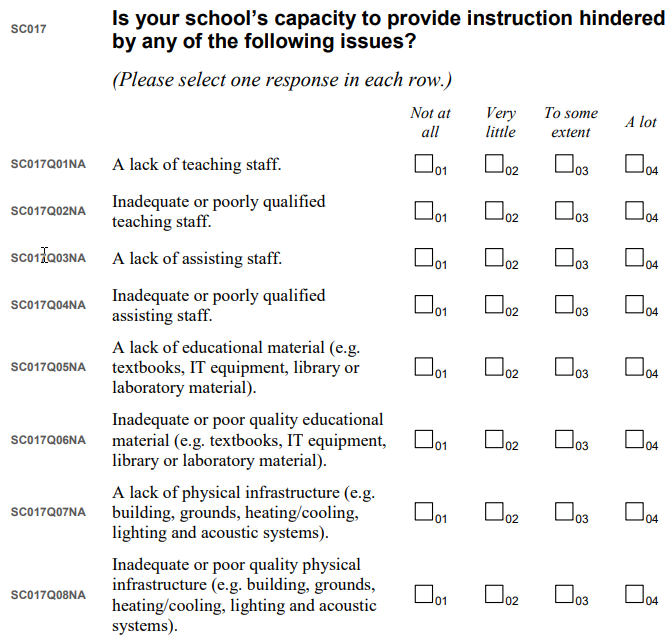


**Appendix B**

R-code to execute the cusp catastrophe model in R using the “cusp” package.

Read Data in R ## if data are in SPSS use haven or foreign packages.

Install cusp package in R ## e.g., install.packages(“cusp”)

Variable names are for asymmetry stub=student behavior, teachb=teacher behavior; for bifurcation sch=school size, clsize=class size.

The syntax lines to produce results are below. After ## there is an explanation for each command.

fit.cusp <- cusp(y ~ readiness, alpha ~ stub+teachb, beta ~ sch+clsize, data = data)

## The above syntax contains the dependent variable y, the asymmetry variables alpha and the bifurcation variables b.

summary(fit.cusp, logist=TRUE)

## The above syntax produces results such as those shown in tables 1 and 2 in the manuscript

plot(fit.cusp)

## The above syntax produces Figures 4 and 6 in the manuscript.

cusp3d(fit.cusp, B=5, n.surf=50, theta=150)

## The above syntax produces Figure 5 in the manuscript
